# Reflections and Methodological Proposals to Treat the Concept of “Information Precision” in Smart Agriculture Practices †

**DOI:** 10.3390/s20102847

**Published:** 2020-05-17

**Authors:** Fabrizio Mazzetto, Raimondo Gallo, Pasqualina Sacco

**Affiliations:** 1 Faculty of Science & Technology, Free University of Bozen/Bolzano, 39100 Bolzano, Italy; raimondo.gallo@unibz.it; 2 Fraunhofer Italia IEC, Bozen/Bolzano, 39100 Bolzano, Italy; pasqualina.sacco@fraunhofer.it

**Keywords:** farm information system, knowledge management, infological approach, automated operational monitoring, certification

## Abstract

Smart Agriculture (SA) is an evolution of Precision Farming (PF). It has technological basis very close to the paradigms of Industry 4.0 (Ind-4.0), so that it is also often referred to as Agriculture 4.0. After the proposal of a brief historical examination that provides a conceptual frame to the above terms, the common aspects of SA and Ind-4.0 are analyzed. These are primarily to be found in the cognitive approaches of Knowledge Management 4.0 (KM4.0, the actual theoretical basis of Ind-4.0), which underlines the need to use Integrated Information Systems (IIS) to manage all the activity areas of any production system. Based upon an infological approach, “raw data” becomes “information” only when useful to (or actually used in) a decision-making process. Thus, an IIS must be always designed according to such a view, and KM4.0 conditions the way of collecting and processing data on farms, together with the “information precision” by which the production system is managed. Such precision needs, on their turn, depend on the hierarchical level and the “Macrodomain of Prevailing Interest” (MPI) related to each decision, where the latter identifies a predominant viewpoint through which a system can be analyzed according to a prevailing purpose. Four main MPIs are here proposed: (1) physical and chemical, (2) biological and ecological, (3) productive and hierarchical, and (4) economic and social. In each MPI, the quality of the knowledge depends on the cognitive level and the maturity of the methodological approaches there achieved. The reliability of information tends to decrease from the first to the fourth MPI; lower the reliability, larger the tolerance margins that a measurement systems must ensure. Some practical examples are then discussed, taking into account some IIS-monitoring solutions of increasing complexity in relation to information integration needs and related data fusion approaches. The analysis concludes with the proposal of new operational indications for the verification and certification of the reliability of the information on the entire decision-making chain.

## 1. Introduction

The first practical experiences related to the application of so-called Precision Farming (PF) technologies date back to the end of the last century and concern the possibility of improving the performance of machines used in spreading or harvesting operations through new automated data management practices. At that time, the paradigm shift in the performance of agricultural activities derived mainly from the ability to associate the quantities of input/output material flows to a specific position of the machines in the field (through appropriate geo-spatial references). On the one hand, this implied overcoming the limits of spatial variability, thus achieving a more detailed and rational knowledge of production sites, on the other hand, the introduction of new problems related to the enormous amount of data to be managed.

These aspects can also be seen from the various additional names under which the PF is still identified today, such as site-specific farm management, target farming, or prescription farming. These terms tend to underline the need to see the PF as a set of technological opportunities aimed at achieving a precise (i.e., spatial) knowledge of field processes, in order to achieve a parallel punctual control of them. In this way, however, the need for “*precision*” advocated by the PF would seem to remain confined to a simple “spatial” concept, which also creates some confusion with the need for “*accuracy*”. Undoubtedly, the technological evolution of positioning systems (with through global satellite navigation systems (GNSS) devices in first position) has played a predominant role in the diffusion of PF techniques. However, the PF is not only GNSS, being able to benefit from all the information and communication technologies (ICT) as a whole [1,2,3]; it has much broader management potentials. An exhaustive definition provided by the US NRC (1997) [4] states that PF is a “*management strategy that uses ICT to collect data from multiple sources in view of their later use in decisions concerning production activities*”. Originally, this definition was firstly focusing on arable farming systems (mainly dealing with cereal crops). Later, it was extended to many other types of farming systems, such as livestock, viticulture, and orchards.

**Figure 1:**
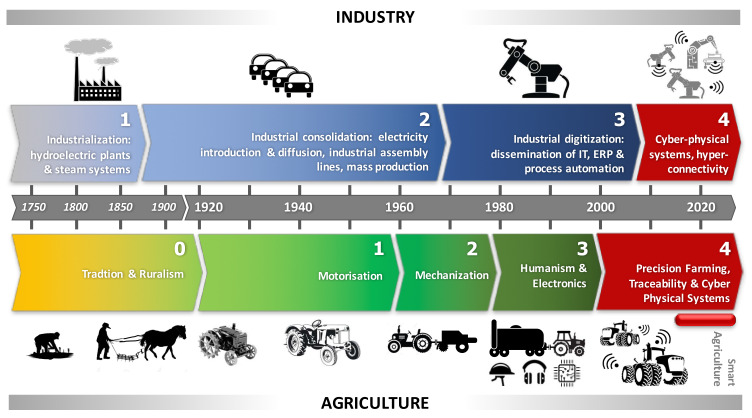
Comparison between the evolutionary technological phases of agriculture and industry.

Despite the relevance of the above original definition, for a long time the PF has been firstly intended as a means to allow the transfer of advanced automated applications into the agricultural sector. This created great confusion on the market among potential users, often disappointed by completely unsatisfied expectations. Only recently also the name “Smart Agriculture” (SA) has started to be used by many producers and researchers in the sector [5,6,7,8]. Frequently, by analogy with Industry 4.0 (Ind-4.0), this term is also substituted by “Agriculture 4.0” (Agr-4.0), sometimes more to reflect a fashion rather than to affirm a new technological principle. As far as Ind-4.0 is concerned, this term is justified by the fact that the industrial sector has been living, for 10 years now, what can be seen as its fourth industrial revolution. For agriculture, a similar approach can only be confirmed if a historical evolutionary vision similar to that proposed in Table 1 is shared (the phases here indicated mainly reflect the Italian situation). A graphical comparison with the technological evolutions occurred in the industrial sector is summarized in Figure 1.

From the analysis of both Table 1 and Figure 1, we must note that a step has been missed in the technological evolution of agriculture. It is the one corresponding to the analogy with the third industrial phase (Ind-3.0, which indicatively occurred in the period 1970–2010), characterized by the introduction of ICT in production processes and the slow but progressive spread of Enterprise Resource Planning (ERP) systems in business management. In other words: in agriculture, a real Information Technology (IT) revolution has been lacking, generally limited to the diffusion of electronic innovations in the Agr-3.0 phase of Table 1, then continued in a more integrated way with the above-mentioned PF logics in the Agr-4.0 phase. From this point of view, the SA can also be seen as an advanced step of Agr-4.0, although the lack of experience and IT tradition in the management of agricultural enterprises is still a brake for a rapid spread of technological innovations in the sector. Obviously, there are also some exceptions: they are all the application domains that come closest—for their structural and organizational characteristics—to the logic of the industrial sector, such as dairy farms, greenhouses, and vegetal nurseries, up to the frontier of the so-called vertical farming.

Despite the above differences, SA and Ind-4.0 share the common paradigm of the so-called Knowledge Management 4.0 (KM4.0), which is based on the need to use integrated information systems (IIS) to manage all areas of any production system [9,10], taking advantage by the irruption of new ICT innovations, such as Internet of Things (IoT), Internet of Everything (IoE) [11,12], Wireless Sensor Networks (WSN), cloud computing, fog computing, and Cyber physical systems (CPS), or a combination of them [13,14,15]. This reference situation for the KM4.0 also requires a paradigm shift in the way data and information are acquired. In fact, here the concept of “measurement” is in practice frequently replaced by the concept of “interpretation”. Information rarely derive from the acquisition of data from a single sensor. Rather, information derive from the ability to quickly integrate raw data from multiple sources. Automated monitoring activities (in their different fields: environmental, productive and operational) often have to take into account a very wide range of aspects, usually resulting from extremely heterogeneous measurements. Monitoring, therefore, is forced to integrate “measurements” through sensors with new devices, such as identification systems and interpretation and diagnosis procedures, often also having to rely on huge amounts of data generated both outside and inside the production system.

There are application areas where these requirements become more relevant. This is the case, for example, of “predictive maintenance” [16,17], fundamental for KM4.0, which constitutes a sort of sub-discipline of data analysis, in which techniques and tools for the development of models capable of predicting future events, failures or behaviors are applied. Prediction models are generally developed based on previous knowledge of the application domain, machine learning (ML) or data mining [18,19]. Through them, we arrive at prediction models with a variable degree of reliability based on the quality of the initial knowledge. The usual concept of “information precision” takes on a different profile, being very often conditioned also by the concomitant execution of a set of interpretation procedures whose reliability depends on aspects not directly related to the quality of the measurements. The classical approaches of pure “metrology”—aimed at assessing how measurement errors affect the parameters of “precision” and “accuracy” of a single measuring apparatus—are no longer predominant, having to deal also with the effects of uncertainty and interpretative quality of the so-called “inference engines”.

It is clear how this situation raises new challenges both to the productive world—which is still struggling to find its way through the various technological opportunities offered by new innovations—and to the research world. This is particularly true for the agricultural and forestry sectors, due to a series of intrinsic difficulties related to the features of their production contexts. The present contribution intends to offer some methodological keys to deal with all these various problems in a conceptual order, maintaining a focus on the quality and reliability of the information that can be obtained from monitoring activities carried out within any SA frame.

This work is, therefore, divided as follows: (i) the first part provides guidance on how to deal with data acquisition from a KM4.0 perspective, taking into account that in any entrepreneurial environment, any choice is conditioned both by the hierarchical order of the decisions to be taken, and by the quality of the a priori knowledge of the nature of the decision-making context. Both of these aspects contribute to frame the concept of “information precision” into a new perspective, replacing the concept of “precision/accuracy of a measure” with the concept of “precision of the decisional context” (=ability to satisfy the needs of a decision maker, according to adequate levels of reliability). (ii) Then, we analyze some explanatory case studies, typical of the agricultural and zootechnical sectors, able to highlight some practical aspects of the theoretical concepts of the first part; they will range from cases of simple measurement devices to more complex situations with integrated acquisition systems in several application sectors. (iii) Finally, we propose possible application strategies to achieve forms of validation and certification of complex monitoring systems, typical of SA contexts.

## 2. Data, Information, and Decision Making Processes

A key aspect of KM4.0 logic is based on the conceptual difference between “raw data” and “information”. The former concerns any type of message derived from the real system of the domain of interest and can be of a quantitative or qualitative nature, as well as be of ordinal or cardinal type. The information, on the other hand, derives from one or more data that can express their usefulness in a decision-making process. It follows that information can be considered an asset with a role both of an input factor (when used to make business/production choices) and of an output factor of the production activity (when used to document business processes or products quality, thus, fulfilling certification and/or traceability functions). The data→information transformation is generally never immediate (with the exception of many automated processes), having to articulate itself through four independent conceptual steps: (A) monitoring, (B) processing, (C) analysis and evaluation*,* and (D) utilization. The development of these steps is made possible through the use of the aforementioned IISs, which act as a sort of digital infrastructure (with hardware and software components), within which all of the transformations of data into information take place, supporting the enterprise’s decision-making and documental processes.

In this regard, specific ontologies have already been proposed [20,21,22] to describe the logical structure of IISs based on the dynamics of data-information transformation. The awareness of such dynamics, easily understandable in appearance, is far from being trivial and obvious. It should address the design of IISs, starting first of all from the identification of the problems to be solved, then fixing the consequent decisions to be taken, then going up to the information that is indispensable to deal with the decisions themselves, and finally reaching the identification of the raw data necessary to generate the necessary information. Using a well-established terminology [23,24], this design procedure is also called “infological approach” (decisions → information → data) and is the opposite of the “datalogical approach” (data → information → decisions), which instead immediately focuses on the need to collect data to identify only at the end the types of decisions that can be addressed with the information they generate.

**Figure 2:**
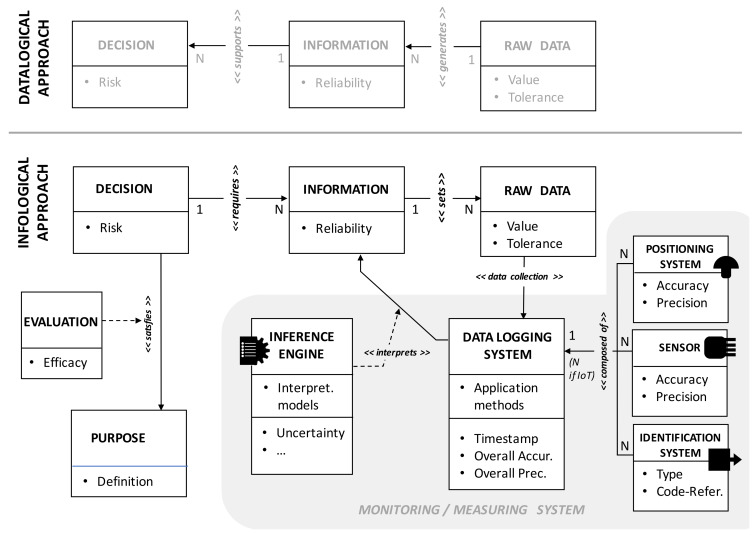
Comparison between infological and datalogical approach and scheme of the decisional chain according to a Knowledge Management logic (KM4.0). The Entity-Relationship graphic formalism based on Unified Modeling Language (UML) is here used. The name of relationships is indicated in the form *<< name >>*. The relationships that implement their own methods are in turn linked (dotted lines) to specific classes (e.g., EVALUATION and INFERENCE ENGINE).

The infological approach privileges the decision-maker’s centrality, while the datalogical approach privileges data centrality (typical in the design of public utility observatories). In a KM4.0 business logic, of course, the design of the IIS should always be set according to the infological approach. However, this is often still ignored (even within the same industrial sector) for various reasons (technical, cultural, commercial), with sometimes serious consequences for the economic-financial and organizational sustainability of the enterprise. The conceptual setting of the two methods is shown in Figure 2, where the role and characteristics of data acquisition systems in monitoring processes are also described.

Here we can observe that:

(1) infological and datalogical approaches share the same main classes of entities (DECISION, INFORMATION, RAW DATA); however, they radically change the relationships between them; in fact, with the datalogical approach, RAW DATA “*generate*” INFORMATION that then “*support*” DECISION; in the infological approach, the DECISIONs “*require*” INFORMATION, which then “*set*” RAW DATA; it is, therefore, a simple conceptual approach which, although virtual, is able to heavily influence the choice of IIS components and its general architecture, with potentially significant repercussions on the quality of the subsequent management of the enterprise;
(2) in the infological approach, the concept of precision depends on the type of decision to be taken and the levels of risk the decision-maker is willing to assume a priori with respect to the efficacy derived from the effects of the decision itself; thus, the quality of decision-making depends on the degree of satisfaction of the objective that the decision is called to resolve (efficacy); from this standpoint, an evaluation process can be represented by a specific class (EVALUATION) that expresses the relationship << *satisfies* >> between the DECISION and PURPOSE entities;
(3) raw data acquisition devices may require the use of different types of basic components (POSITIONING SYSTEMS, SENSORS, IDENTIFICATION SYSTEMS) that are often to be included in integrated combinations; each component has its own application methods and helps to condition the information reliability through the quality of the measures it can perform (conditioned by its precision and accuracy attributes, which must satisfy the tolerance requirements of the raw data);
(4) data acquisition also has its own temporal dimension, of fundamental importance to reconstruct, then, the dynamic aspects of production processes; generally, the timestamp is in charge of the DATA LOGGING SYSTEM, which also performs synchronization (and eventually integration) functions with respect to the signal acquisition frequencies of each basic component attached to it;
(5) the reliability of the transformation of raw data into information depends, besides on the instrumental reliability, often also on the need for interpretation of information through appropriate algorithms supported by inference engines; these procedures introduce a further degree of uncertainty that will ultimately affect the quality of the decision to be taken, with effects on the related levels of risk;
(6) the above mentioned basic components usually have a one-to-many (1:N) relationship with the DATA LOGGING SYSTEM (“*composed of*”); the innovations introduced with the IoT and IoS solutions have allowed to modify it in a many-to-many (N:N), thanks, above all, to the possibility to manage the connections remotely, and to use specific communication protocols, including solutions based on decentralized architectures, such as cloud/fog computing. If on the one hand this allows to have undoubted advantages, thanks to a greater constructive simplicity and management flexibility, on the other hand it involves less customization of the components themselves, and a different attention in the use of INFERENCE ENGINE, which will have to be redefined according to the needs of each specific domain of interest. The world of research will be destined to develop relevant insights in this field, especially in the fields of application of the already mentioned “predictive maintenance”, where developments are expected especially in terms of interpretative skills of IIS (Machine Learning).

All considered, when operating within production systems; thus, going beyond the typical applications of the research or certification world, where the main focus of the reliability of the measures mainly concerns instrumental devices, the common concept of “information precision” can never be intended in absolute terms, rather, it must always be placed in relation to the nature of the decision to be taken and the risks that may derive from the poor quality of the latter.

## 3. Type of Decisions and Quality of Monitoring

### 3.1. Hierarchy of Decision-Making Levels

An enterprise is an organization structured on several hierarchical levels, where strategic, managerial, and operational decisions exist, depending on the purpose of the decision-maker involved. The KM4.0 approach, which privileges a management vision based on the centrality of information, sees an IIS, first of all, as a system in which information must easily flow through the various hierarchical levels to facilitate decision-making processes. The information flows generated at operational level must be transformed, combined, and consolidated before reaching the management level. Here they will be further processed and aggregated before returning to operational or strategic levels. In general, it is precisely the intermediate “managerial” level that is in charge of the greatest workload in data processing and information production through processes of: (a) strategic summary: when intended for the person in charge of long-term planning activities (entrepreneur); (b) operational summary: when intended for the executors of the individual basic processes (e.g., field workers and tractor drivers).

**Figure 3:**
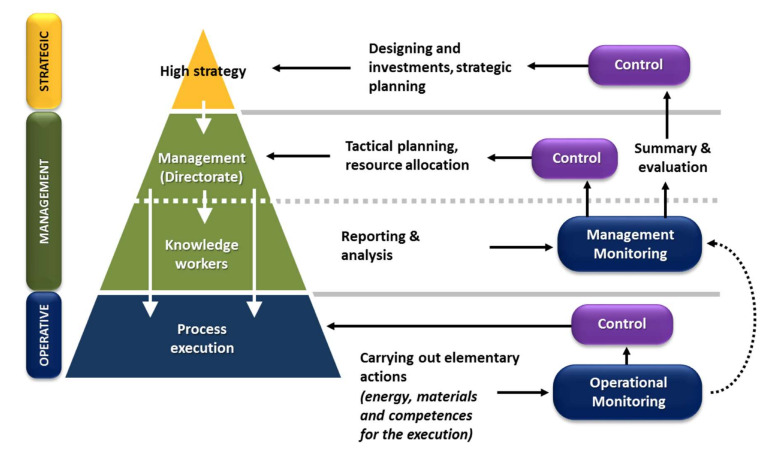
Schematization of the hierarchical levels of an enterprise and indication of the main tasks of the responsible people at each level (derived from [27]).

This generally leads to an overburdening of the intellectual workload for the people involved at the management level that, necessarily, must be able to rely on competent and responsible staff. This is a problem common to all production structures: in the industrial and tertiary sectors, the progressive digitalization of company procedures has now made essential to have a new professional figure to support the actual company management. These are the so-called “knowledge workers” [25,26], whose role is to: (i) coordinate and manage the collection of company data; (ii) carry out intermediate processing and analysis with the production of summary information; (iii) provide for the production of documentation and the distribution of information in the various company sectors, to put into practice the decision-making processes of the management. The overall description of the company hierarchical structure can therefore be represented through the scheme of Figure 3, which extends the already well-known formalism proposed by Anthony [27,28]. The scheme highlights the main functions typical of each sector, together with the fact that each hierarchic level performs its own type of control activities (execution control, management control, high strategy).

The term “control” is here intended as a set of observing actions (through monitoring activities) and verification tasks with the aim of regulation, orientation, and dominion over the different types of activities foreseen in the production system. Thus, each control activity then involves an intervention, i.e., an immediate or deferred action-on the production system. In the case of operational decisions, the actions are normally immediate and consequent to process controls, environmental controls (generally always in support of process controls), and product controls. In the case of tactical decisions, on the other hand, the actions are generally deferred (planning) and are carried out through resource management interventions (coordination of personnel, allocation of technologies and materials), always aimed at both process and product controls, often also functional to certification objectives (documentation and traceability).

### 3.2. Quality of the “A Priori Knowledge” and Domains of Interest

KM4.0 approaches could offer valuable solutions to the simplification of knowledge workers’ roles. In fact, both the IoT techniques and, above all, the opportunities of the IoS make us hope for the spread of new generations of IISs with partial management in charge of external service centers, which will be able to support specific aspects of production processes mainly through remote connections, also based on decentralized cloud/fog architectures. In order to achieve this, the margins of reliability, interpretation, and diagnostics of the Inference Engines involved in the monitoring activities must be enhanced as much as possible. This, once again, brings the problem back to the margins of reliability and tolerance to be attributed to data acquisition systems. For all of the above, reliability and tolerance tend to depend on:

(1) the decision-making level and purposes of the decision-maker;
(2) the quality of the “a priori knowledge” that features the control activities required by the decision-making process, a quality that in turn depends on the features of the reference application context (domain of interest).

In the domains of interest of the industrial sector—due to the repetitiveness of the processes, the high uniformity of the products, and the circumscribed and controllable production environments—process and product controls (mainly based on the use of conventional standard measuring devices) prevail, with the tendency to make the concept of reliability of the production system coincide with that of reliability (precision/accuracy) of the means of production and the relative control (and measurement) instruments. The domains of interest of the agricultural sector, on the other hand, include production systems with elements of more difficult control, typical of the environmental and biological components. Here, environmental control becomes a primary task, which is difficult to achieve given the variability and extension of production contexts; moreover, the strict uniformity of primary products is practically impossible to achieve, due to the intrinsic variability of the “living” means of production.

Even in the industrial sector, however, there are areas of application in which the “a priori knowledge” of control activities takes on a fuzzier connotation, increasing the margins of uncertainty in decisions. Again, this is the case of “predictive maintenance” functions in place of “planned maintenance” ones. For the latter, it is sufficient to keep updated the interventions and timing of a work plan (fixed in advance). For the former, on the other hand, it is necessary both to set up measuring devices to guarantee continuous monitoring and to develop “forecasting” instruments that must be able to anticipate with reasonable reliability the behavior of components subjected to different types of functional stress [17,19]).

### 3.3. Macrodomains of Prevailing Interest

To better understand the levels of difficulty associated with the different types of elements that condition a production system, it is useful to refer to the concepts of the theory of systems, according to which any system is composed of an observable part (of which the knowledge explaining structural and behavioral aspects is fully and exhaustively known) and an unobservable part (characterized by knowledge that is still incomplete and imperfect). The larger the observable part is when compared to the unobservable part, the more relevant will be the controllable part of the system compared to the uncontrollable part [29].

Although simplified, this schematization has very important practical implications in the setting up of data acquisition systems, especially in their part concerning the reliability of the inference engine, if any. In fact, when the observable part clearly prevails over the unobservable part, we are dealing with a system of perfect knowledge that does not set technological limits to the degree of reliability that—depending on the objectives—is intended to ensure the data to be acquired and the quality of subsequent information that may derive from it. On the other hand, when the non-observable part clearly prevails over the observable part, there are no proven theories that can condition a containment of the degree of uncertainty about the information to be acquired.

According to this vision, it is possible to introduce the concept of “Macrodomain of Prevailing Interest” (MPI). An MPI identifies a specific point of view by which a system can be analyzed according to a prevailing purpose. The quality of the analysis depends on the cognitive level and its related methodological approaches permitted by the knowledge maturity reached by the MPI itself (i.e., observable vs. not observable part [29,30]).

**Figure 4:**
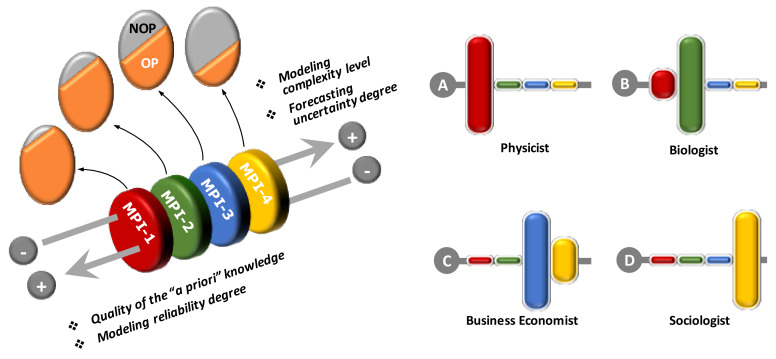
Schematic approach to describe the definition of Macrodomain of Prevailing Interest (MPI). Left side: a system can be seen as divided into two parts: observable (OP, orange) and unobservable (NOP, grey). The larger the observable part, the higher the quality and details of the knowledge we have of a system, with a direct effect on the quality of the information that affects the decision-making capacity on the system itself. Four different information quality levels can be identified, corresponding to the four MPIs described in the text. Right side: the qualitative decision-making profile of a decision-maker can be then described as a combination of different MPIs; some examples of “pure” professional standpoints are here provided.

As qualitatively described in Figure 4, it is possible to classify four main MPIs, according to a decreasing level of the observable/controllable part (OP vs. NOP):

- **MPI-1**: **Physical and Chemical MPIs**, in which exact sciences and well defined/proved theories prevail;
- **MPI-2**: **Biological and Ecological MPIs**, partly yet conditioned by empirical sciences, with some theories that must be further improved, typically conditioned by the still fuzzy knowledge of some aspects of life sciences;
- **MPI-3**: **Productive and Hierarchical MPIs**, largely conditioned by empirical sciences, since also dependent on the cognitive behavior of single individuals; they require direct observations and analysis of trends;
- **MPI-4**: **Economic and Social MPIs**, concern complex systems in which the behavior of large masses of individuals prevails (contexts of not-exact sciences); the main investigative tools relate to economic and social observatories, market surveys and statistical sciences.

Every decision-maker (be he a scientist, a technician, a business manager, a worker, or a consultant) looks at his domain of interest through a point of view that depends on the relative weight he assigns to the various MPIs in conceptually modelling an abstract representation of the real system under examination. Thus, for example, for a physics researcher the domain of interest will always be modelled through the a priori knowledge of MPI-1 only, while for a pure biologist the domain of MPI-2 will tend to prevail (although supported by the basic knowledge of MPI-1), for a sociologist or politician almost exclusively MPI-4 will prevail. Apart from some extreme specializations, the point of view of many professional figures will result as a mixture of competences related to the various MPIs, although generally a specific MPI always tends to prevail over the others, influencing the levels of knowledge quality of the overall decisional profile.

Ultimately, the combination of the four MPIs defines the point of view of the decision maker and conditions the levels of reliability and tolerance in his decision-making process (as always qualitatively shown in Figure 4).

**Figure 5:**
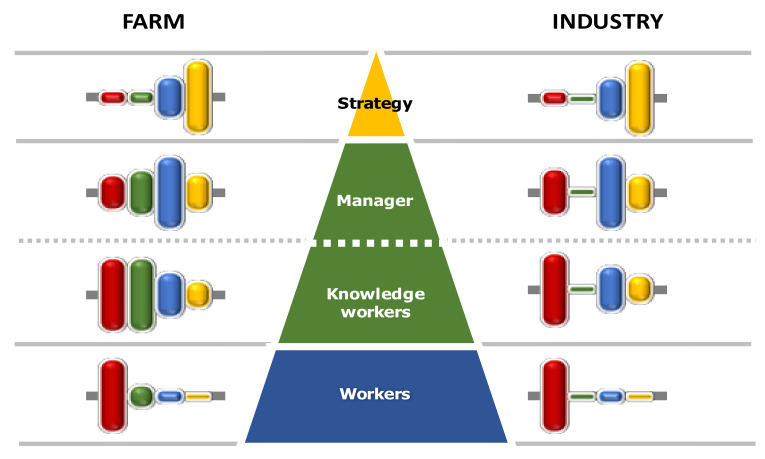
Probable combination of MPIs in the different hierarchic levels for agricultural and industrial enterprises.

For both SA and Ind-4.0, the main domains of interest concern areas under the MPI-3. However, while for Ind-4.0 the detailed constituent elements are mainly concentrated in the MPI-1, the components of the MPI-2 prevail in the case of SA. Here, the biological and environmental elements largely condition the reliability of the information and the degree of uncertainty in the decisions, and require that the interpretative models often assume a preponderant role in the measures (monitoring and control activities). Obviously, the role played in the enterprise’s hierarchy also takes on considerable importance in outlining the overall decision-making profile, as briefly described in Figure 5.

**Figure 6:**
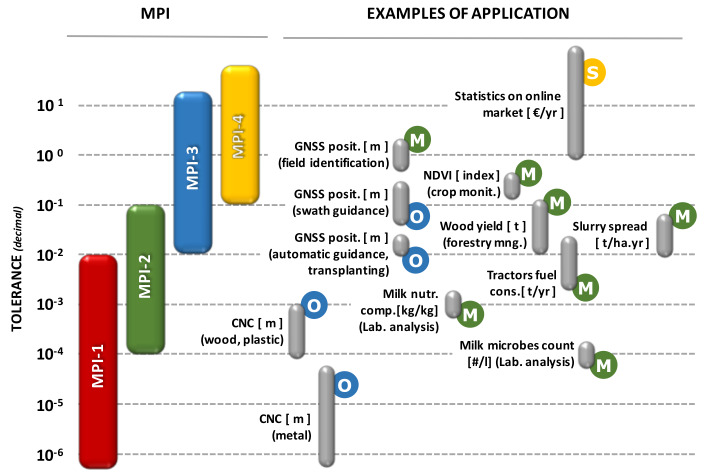
Typical ranges of tolerance in the four MPI (Macro-domains of Prevailing Interest, refer to Figure 3) and some examples of application. The usual hierarchic decisional level is also indicated (O: operative, M: management; S: strategic). Global Navigation Satellite System (GNSS); Computer Numerical Control (CNC).

In conclusion, owing to the different degrees of uncertainty resulting from the mix of the four MPIs, the tolerances allowed for the information (and consequently for the measuring equipment used to acquire it) may vary as described in Figure 6, where some practical application examples are also given, together with the related decisional level usually involved. The tolerance is there indicated as a decimal point to be referred to the unit of measurement of each example case.

It is possible to observe that:

1. Computer Numerical Control (CNC) machines, typical of many industrial processes, are among the means of production that allow the lowest tolerances (up to 10^−6^ m), however, with a very wide range of variability depending on the process and the type of material being processed. Here we operate in the full domain of MPI-1;
2. there are needs for information that-regardless of the decision-making level that requires them (operational or managerial)-admit very narrow tolerance ranges; in the examples considered here, they concern both the automatic guidance rather than the fully automated field transplantation operations (MPI-1), and the feedback from laboratory tests aimed at a product control (MPI-1 or MPI-2);
3. measurements for information related to Management Support Systems (MSS) applications generally allow higher tolerances, especially when the final information results from the aggregation of several measurements (e.g., yearly fuel consumption) or the integration of several measurement systems used in continuous monitoring activities (e.g., slurry spreading); in the SA applications—as well as in many predictive maintenance tasks—the information provided by an IIS often derive from a very articulated combination of monitoring and measurement systems between elements referable to different MPIs, whose aggregation generally involves significant phenomena of error propagation with a consequent increase in the degree of uncertainty;
4. the information for Strategic Decision Support Systems (SDSS) are those characterized by the highest tolerances. Unlike the other cases, in fact, here the information often also comes from observations carried out outside the production system (e.g., average costs/prices of production factors, market analysis, statistics on consumer trends, etc.). In this way, the acquisition procedures outlined in Figure 2 are avoided, given that data logging systems are usually replaced by database access tools, by the use of simulation models for the analysis of hypothetical alternative scenarios, or by the carrying out of surveys through sample interviews; in this case we work on elements typically related to MPI-4.

In case of lack of data, anyhow, the use of conceptual tools other than a data-logger system (database, simulation models, direct experience of the decision-maker) may be applied in all decision-making levels, not only in the strategic one. This is evident considering the impossibility to submit all aspects of the real system to continuous monitoring processes. Obviously, they introduce elements of uncertainty, with related consequences on the information reliability, similar to those of an inference engine. Such reliability will be all the more limited the more the quality of knowledge incorporated in these “alternative tools” (including the experience of the decision-maker) can rely on the solid foundations of MPI-1 and MPI-2.

## 4. Analysis of Some Practical Examples

**Figure 7:**
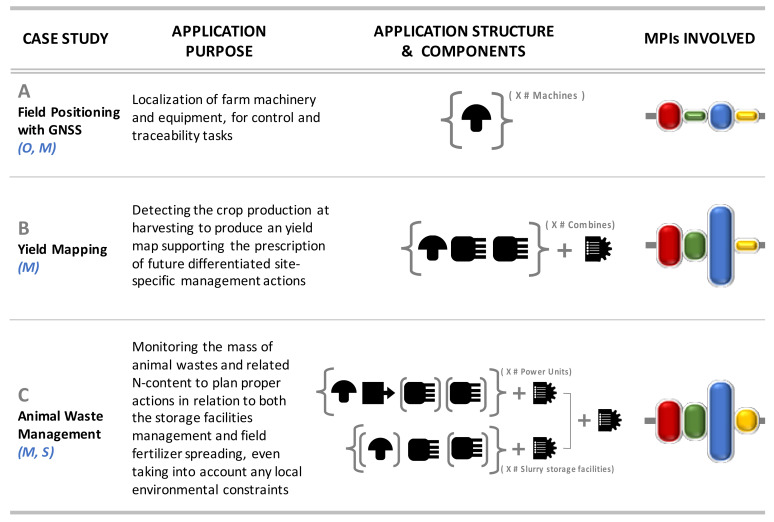
Summary of the case studies discussed here below. For component symbols, refer to Figure 2. Brackets “{ }” indicate whole data acquisition systems to be installed in quantities equal to the number of agents (tractors, combines, storage units etc.) to be monitored at the farm. Parenthesis “( )” indicate optional components (in case C, e.g., they can refer to sensors for detecting the tractor engine rpm or slurry electrochemical conductivity to provide more monitoring details in case of Field Operation Register Keeping (FORK) or slurry storage units, respectively.

The agricultural sector provides a variety of application examples relevant to the aforementioned methodological considerations. The case studies proposed here are summarized in Figure 7, where the synthetic descriptions of the data acquisition systems (with related components) proposed in each case are also reported.

The first case (A) concerns satellite positioning systems, chosen since they are used as components in many SA applications. Being a “single component”, the focus is on how to evaluate their performance (according to the classic criteria of “accuracy” and “precision”), whose tolerances depend on the application purposes in the farm. Thus, the quality of knowledge involved in their applications concerns aspects limited to MPI-1 and MPI-3.

The second case (B) concerns, instead, the realization of data acquisition systems for the generation of yield maps, conceived as a set of components to be installed on board a single carrier (usually cereal combines). The collected data are then integrated by an inference engine (operating usually-but not necessarily-in post-processing) that will provide, finally, to generate thematic maps to be used as support in subsequent decision-making processes (e.g., fertilization plans). Here, obviously, the aspects of MPI-3 prevail, supported by the knowledge of MPI-1 (e.g., operation of sensors and GNSS receiver; mechanical and kinematic behavior of the combine) and MPI-2 (e.g., physiological and morphological aspects of the variety, also in relation to soil types; variability of the canopy), with levels of reliability and tolerance of the final information (=geo-localized estimate of dry matter production), generally between 2% and 5% with respect to the expected real values, but with peaks even until 10% in the most unfavorable conditions (field <2 ha, with irregular shape) [31]. In fact, in these applications, in addition to having a geometric propagation of instrumental errors, complex aspects typical of the “uncontrollable” part of the MPI-3 domains are involved, such as the driving modes of the machines, with behaviors that then also affect the performance of the other single components.

Finally, the third case study (C) concerns an example of integration between several independent data acquisition systems. This is the integral monitoring of waste management systems in animal farms, which includes biomass balances extended to both storage systems and spreading modes of organic fertilizers in the fields. The overall decision quality profile is similar to case B, with the extension to aspects of MPI-4 due to the possible need to apply these systems to meet some specific environmental regulations.

### 4.1. Field Positioning of Farm Machinery and Equipment

The introduction of affordable solutions for the positioning of machinery through global satellite navigation systems (GNSS) has probably represented the technological innovation that more than others has favored the spread of PF techniques in different global contexts [32,33]. This is undoubtedly due to the ease and flexibility of use of GNSS receivers, which can be easily integrated into machinery and adapted to any applicative situation, without the need of creating expensive external supporting infrastructures [34]. However, for some time, the discussion on the performance in terms of accuracy and precision of these devices has involved different areas of research in various sectors being expanded, with (negative) repercussions of some significance also at the commercial level. In Italy, towards the end of the last century, swath guidance devices were still marketed at prices ranging from 15% to 25% of the new value of a tractor; the same was true for positioning devices used for cereal yield mapping (with peaks of up to 30% of the new value of combine harvesters) [35].

This has created situations that are decidedly unsustainable, due to many causes, first of all the proposal of expensive solutions for differential corrections (DGNSS), often useless (and difficult to manage) for the specific applications proposed, which certainly did not require solutions capable of ensuring extreme levels of precision and accuracy. Since the first decade of this century, many attempts have been applied to make order in this sector, with the aim of establishing minimum safety and precision requirements according to the field of application, which-especially in the agro-forestry sector-has very different technical and management purposes.

**Figure 8:**
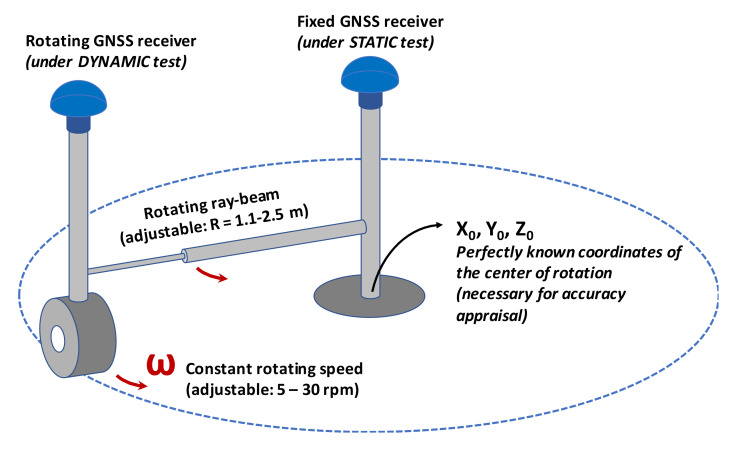
Scheme of a “RotoGPS” used for performing contextually both static and dynamic test on the same type of two GNSS receivers. A measure of both accuracy and precision of the receiver at hand is simultaneously achieved for the two test conditions.

**Figure 9:**
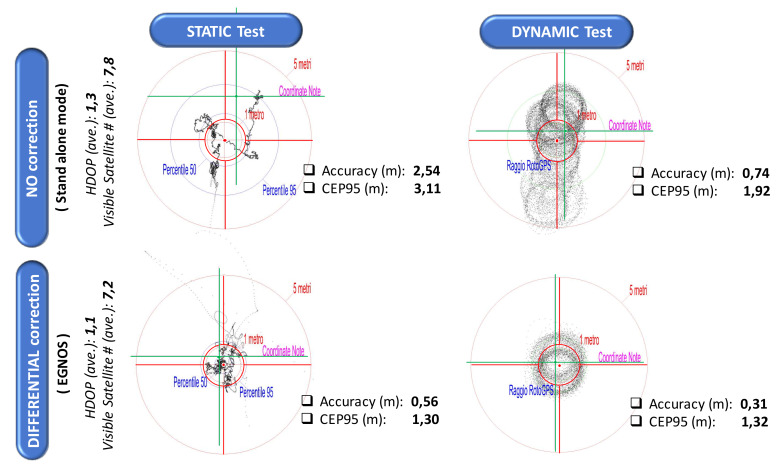
Examples of static and dynamic tests simultaneously carried out with the device described in Figure 8. The tested receiver is a good performance model that can be used both in a stand-alone mode and with a real-time EGNOS differential correction. The green cross axes indicate the actual position of the measuring system, previously geolocalized with extreme accuracy. The red cross axes (with the central circle indicating the expected trajectory of the rotating arm, here 1.2 m long) refer to the medium performances around the centroid of the overall fixings (10800, after 3-h test). CEP95: radius of the Circle Error Probable, where 95% of the fixings would fall in; HDOP: Horizontal Dilution Of Precision

This has also favored the development of testing and certification systems capable of ascertaining the performance of the various types of receivers not only through traditional static tests over extended periods of time, but also through dynamic tests, much more adherent to the types of applications of agriculture. Worthy of mention in this regard is the so-called “RotoGPS”, a device capable of testing in a single solution two identical receivers, one fixed on a center of rotation and the other rotating at a constant speed at the distal point of a rotating ray-beam (Figure 8) [36,37]. In this way, the device is able to provide simultaneous measurements of accuracy and precision, in both static and dynamic operating conditions. An example of the results that can be obtained with it are shown in Figure 9.

They relate to a good performance commercial receiver that can be used in both stand-alone and differential correction conditions provided by the EGNOS network. The accuracy is quantified here as the distance between the centroid of the points detected during three continuous hours of operation (here 10,800 fixings) and the point corresponding to the RotoGPS pivot pin (with coordinates [Xo,Yo] in Figure 8), while the CEP95 parameter (radius of the Circle Error Probable, where 95% of the fixings would fall in) is used to estimate the precision. In the figure, the pivot pin is located at the intersection of the axes plotted in green, while the centroid is located at the intersection of the red axes (combined with a red circle indicating the expected trajectory of the rotating point, here with a radius of 1.2 m). Analyzing the results, we can observe that:

1. in static tests, the position drift observable in the time sequence of fixings follows apparently random trajectories, mainly due to the arrangement of the satellite constellation visible from the receiver; every time the constellation changes there are variations in position, which in some cases can also be extremely significant despite the presence of a differential correction;
2. in dynamic tests, the phenomena related to constellation variations are more evident since they have the effect of translating the “virtual” center of the rotation pivot in different points, with evidence of the permanence of different circular trajectories;
3. as usually happens for many receiver models, the performance in dynamic tests tends to improve when compared to static tests; this is true for both accuracy and CEP95 (precision), given the geometrical effects of retracing the same trajectory several times; this aspect takes into greater consideration a behavior similar to that of many agricultural operations that process the same space even through adjacent parallel passes in sufficiently short time periods (e.g., swath guidance);
4. the positive effects of the differential correction are very visible, also in this case both on accuracy and precision; the improvement is especially evident in the case of dynamic tests: with an accuracy that goes from 0.74 to 0.31 m and a CEP95 that varies from 1.95 to 1.32 m the overall result in the three hours of testing is very close—including visually—to the expected theoretical trajectory, with a considerable mitigation of the effects related to constellation changes;
5. just for the effects related to the characteristics of the satellite constellation available during the tests, these should be performed only when the expected horizontal diluition of precision is HDOP < 2 (this condition, however, is easily verifiable a priori).

**Figure 10:**
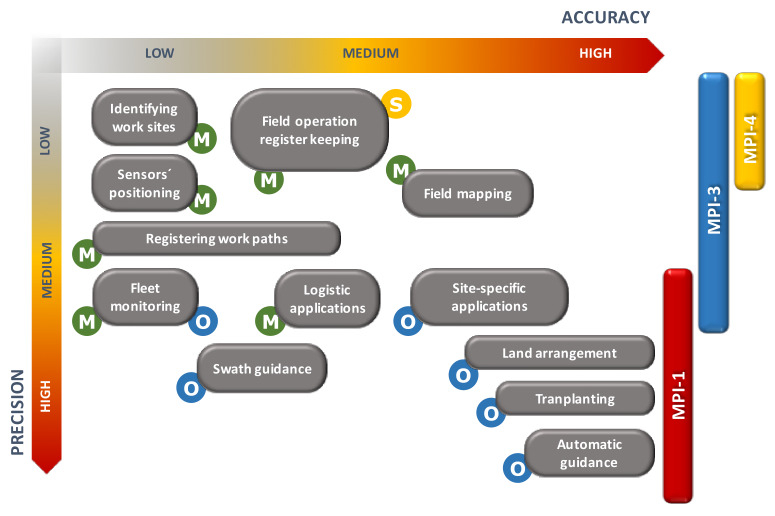
Accuracy and precision requirements for GNSS receivers to be used in different agricultural applications. The main decision-making levels and MPIs involved are also indicated. For the symbols, refer to Figure 6. As quantitative reference, the following range values can be considered: (**a**) LOW: >20 m; (**b**) MEDIUM: 2–5 m; (**c**) HIGH: <0.05 m [36].

Efforts should be further increased in this sector to promote standard certification procedures capable of qualifying performance also in relation to a particular application function, always focusing on the objects of the prevailing MPI and the type of decision-making process there supported, as indicated in Figure 10.

Mostly, we have to deal with operational and directive decisions, mainly referring, respectively, to the macrodomains MPI-1 (with many applications in the field of automation of field processes) and MPI-3 (with various applications in the field of operational monitoring). There are, however, also possible references to MPI-4, especially in the case of mandatory controls that refer to explicit regulations with territorial management constraints (e.g., verification of the spreading limits of N-loads or the need for objective evidence of the delivery of products at specific functional sites).

For some applications, the nature of the decision-making level involved can also be twofold, depending on the specific objectives to be achieved. This is the case for various applications of operational monitoring: the FORK systems (Field Operation Register Keeping, [22,38]) usually have a predominantly managerial profile, as they can be used in the optimized planning of seasonal activities in the field, in the weekly organization of the work chains or in the control of the correct performance of current activities. However, they could also be used in a product certification approach for the maintenance of high quality standards over the long term (e.g., to demonstrate detailed adherence to particular production specifications), thus bringing them closer to strategic targets (with MPI-4 requirements). Similarly, in fleet monitoring applications, operational or management objectives may prevail depending on whether the need for real-time control over the location of the vehicles on the territory prevails, rather than ex-post verification of the sites (or fields) visited (or treated) over a given period of time.

Whatever the case, in all these applications low accuracies (>20 m, often compensated by the dynamic trajectories of the machines in the field) and medium-low precision (5–10 m) may be sufficient, with relative savings on the cost of the measuring devices. High accuracy/precision receivers (<0.05 m) should be limited just to highly automated operations subjected to strict spatial constraints [39,40]. It would be advisable to achieve, with a single testing methodology (such as RotoGPS, through both static and dynamic measurements), a certification of the performance of satellite receivers with their evaluation of merit with respect to the various application classes that can be outlined according to a scheme similar to that shown in Figure 9.

### 4.2. Yield Mapping

The production of cereal yield maps represents one of the first (pioneering) major technological innovations in PF because of their potential usefulness in crop management decisions. For many years, it has been seen as a sort of “symbolic” application for PF, a real reference technology for this sector [41,42]. Its pragmatic application in decision-making processes, however, has always led to numerous problems:

- from a cognitive point of view, it presupposes the downstream availability of a series of technologies at farm able to implement site-specific control interventions, consistent with the level of detail provided by these maps (e.g., differentiated soil tillage management, site-specific dosage of fertilizers and agrochemicals); such availability is not always verified, with consequent frustration of the perceived usefulness of the information provided by the yield maps;
- from a technological point of view, the data acquisition system that allows the realization of yield maps has represented for PF a first challenge of data fusion and integration of interpretative methods, with relevant implementation problems for constructors. Such difficulties have been always few perceived by farmers, as the latter are only focused in the prompt availability of a thematic map [43].

The first point refers to the difficulties of creating complete PF supply chains in farms, especially if connected to information systems designed according to KM4.0 logic. To this end, it would be useful to see the decision-making usefulness of yield control at least at three different levels: (a) long-term decisions, considering strategies such as crop rotation and yield stability over time, (b) intermediate decisions, related to the upcoming growing season, varietal selections, fertilization and phytosanitary treatment planning, and (c) short-term decisions, related to crop management in current soil and weather conditions, up to the formulation of prescriptive maps for the site-specific implementation of fertilization and pesticide distribution. These are, therefore, decision-making levels of both directive-management type (which extend to the generation of prescription maps with the help of knowledge workers) and operational type (limited to the execution of fertilization and chemical treatments).

The second point refers, instead, to technological problems of the data acquisition system, not yet completely solved, which involve the contextual presence of the following types of errors in the realization of yield maps [44,45]:

(a) positioning by GNSS;
(b) indirect instrumental measurement of grain flow (e.g., with sensor counting impact pulses on a metal intercepting surface);
(c) indirect measurement of grain moisture (e.g., with capacitive sensors);
(d) spatial synchronization between positioning data and flow data (depending on the construction features of the combine harvester, its working speed and the dynamics of transport of materials within it) [46];
(e) projection of the data of a single fixing on a given surface (geostatistical elaboration, variable according to the intention to proceed with iso-production areas or with mosaic of cells of predefined shape and size) [47].

The consequence of this is that mapping errors are largely conditioned also by the surface of the worked fields. They can be acceptable or negligible (<2%) in case of correctly calibrated systems, operating on regular shape fields over 10 ha; they become critical and unacceptable (>10%) in case of non-calibrated systems and on very irregular shape fields of small size (<2 ha) [31,48]). Yield monitoring has certainly reached a consolidated level of technological maturity, so much so that all the major manufacturers of combines offer proprietary standard solutions that try to limit, as much as possible, the above problems, mainly through regulation systems designed to promote better synchronization between the reading of grain flows and the related collection points on the fields (lag time settings, presence of a header position sensor to detect the real cutting phases), as well as awareness campaigns for frequent sensor calibration through on-farm interventions [43]. Despite this, the problems of reliability of the measurements are not yet completely solved and the research for improvement in this sector has always continued [49,50].

Ultimately, to assess their reliability, these maps should be accompanied by a parallel *map of tolerance verification*. This should provide indications on the reliability of the measuring system by geolocating the tolerance and reliability of the final estimates on standard reference parcels (possibly of various shapes) and under different harvesting operating conditions (e.g., forward speed, output flow rate, accuracy/precision class of the GNSS used, geostatistical method applied with related setting parameters).

This is also advisable, considering that yield mapping is now extended to a wide range of cropping systems (including viticulture) and that more and more external consulting services will have to be used both for the elaboration/interpretation of yield maps and for their integration on multi-layer GIS platforms aimed at the redaction of prescriptive maps (thus replacing the internal role of knowledge workers) [51]. It should be obvious that cartographic integrations of thematic maps are reliable only when each map proposes comparable levels of detail, reliability and tolerance. The lack of compliance with this requirement risks frustrating the effectiveness of these analytical initiatives. In the application domain of these integrated solutions, the aspects of MPI-1 and MPI-2 have considerable weight. They must be anyhow adjusted to the decisional objectives of MPI-3, aiming at reliable yield assessment for planning future decisions.

### 4.3. Animal Waste Management

This is a management problem of greater complexity that includes different decisional objectives of a livestock farming system, usually not addressed by a simple measure, but by the organization of an internal network of operational monitoring concerning the functions of different production sectors interacting with each other [52,53]. In general, these objectives include: (i) control of the quantity and quality of the biomass in the storage facilities; (ii) keeping of the field spreading registers, with the relative production of reports for the traceability of the activities actually carried out [54,55,56]; (iii) production of transport documents; (iv) continuous verification of the nitrogen loads actually distributed during the year and compliance with the limits imposed by the current environmental regulations.

According to the logic of KM4.0, all operational monitoring activities to be implemented must meet the needs of automating both data collection and successive interpretation of information acquired, possibly following approaches of connectivity and integration among the various sectors involved. In practical terms, the following should be kept under continuous observation: (a) the amount of animal wastes in storage facilities; (b) the concentrations of nutrients in wastes (in particular nitrogen), and (c) any activity relating animal waste handling (loading and unloading of storage, field spreading). In case of liquid waste (slurry), the monitoring activity implies a measurement of the level reached by the biomass periodically uploaded in the storage facilities.

**Figure 11:**
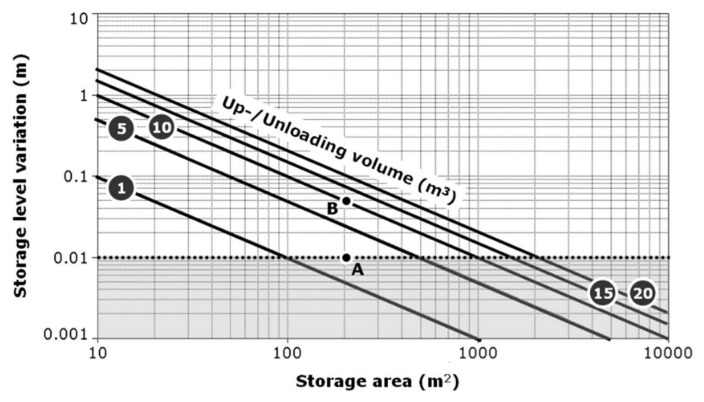
Sensibility of storage level variations vs. storage surface and the effluent volume to be manipulated. Grey area indicates conditions that are out of the control of the monitoring system, being practically impossible to get slurry level measurement with resolutions lower than 1 cm. The two points A and B refer to a 200 m^2^-surface slurry storage located below the holed floor of the stable where 2 m^3^/day of wastes are on average produced by the herd (A) and a 10 m^3^ slurry tank is used for spreading (B). Only the event B can be usefully detected being greater enough than the sensibility of the monitoring system.

**Figure 12:**
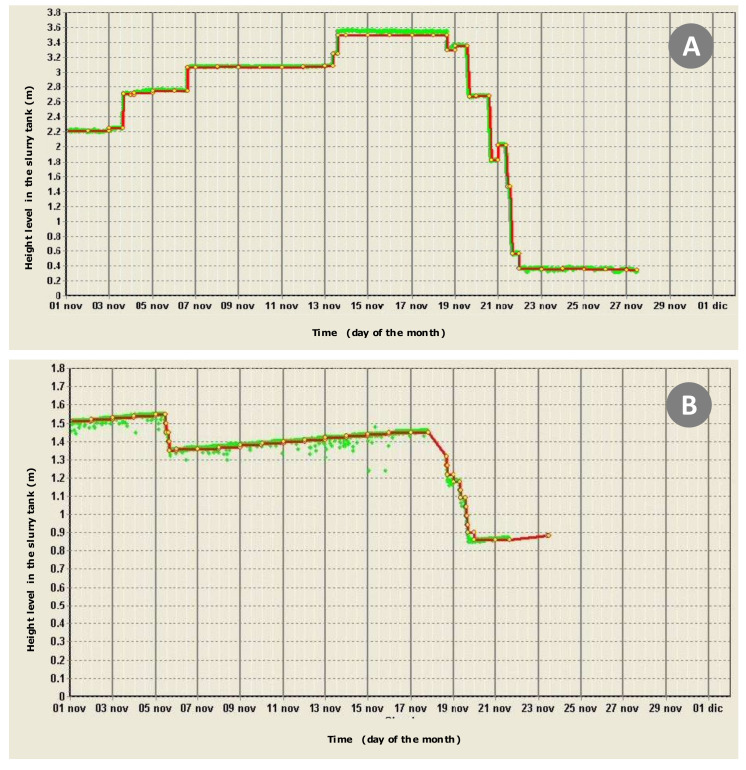
Examples of monthly slurry level monitoring results on two different storage structures (logging frequency: 6 h^−1^). An inference engine converts raw data (green points) into stable level benchmarks (red lines). (**A**) Aboveground cylindrical slurry tank (surface: 350 m^2^). (**B**) Parallelepiped-shaped slurry tank (220 m^2^) placed under the dotted floor of the stable with freestanding animals; being continuous, here, upload events are not detected (progressive increase of the slurry level).

Measuring a level may appear to be a very simple problem from a conceptual point of view, typically confined to aspects of MPI-1. However, in practical terms, its implementation for management purposes (thus, framing its application in an MPI-3 framework) is faced with some problems that require careful attention. In fact, we have that:

1. the measurement can be carried out in different ways, for example using pressure or ultrasonic sensors, to be placed, respectively, on the bottom of the tank or at a certain height with respect to the surface reachable from the maximum level; pressure sensors provide stable measurements over time, but involve serious maintenance problems being immersed in a rather corrosive environment; ultrasonic sensors have opposite features, with the addition of noises in measurements due to undesirable phenomena (uneven surface, often moved by the convective phenomena of fermentation, as well as subject to the growth of vegetation on the surface);
2. level variations depend on the free area exposed in the storage facilities and the volumes transferred during each loading/unloading operation; the relationships among these three parameters is shown in Figure 11; here, it is highlighted that the acceptable tolerance threshold value for the level measurement provided by the afore mentioned sensors is theoretically around 1 cm; instruments with wider tolerances would be not very effective compared to the objectives of the control system; however, even with acceptable tolerances (2–3 cm), the measurements are generally affected by a significant noise, which makes it impossible to simply read values averaged or interpolated with conventional statistical methods; in this case, therefore, it becomes necessary to use an inference engine in order to fix the levels around stabilized reference values (benchmark levels) through signal analysis algorithms [57];
3. according to Figure 11, shape and size of storage facilities directly affect the performance of monitoring activities; generally, aboveground cylindrical tanks with volumes up to 3000 m^3^ are able to highlight loading and unloading events with sufficient clarity. Conversely, parallelepiped-shaped slurry tanks placed under the grating floors of the stables with free-standing animals, are subject to a filling that takes place gradually all along the day; given their high extension (often >500 m^2^), the monitoring system only detects a progressive increase in levels, which can only be assessed over sufficiently long periods of time. Examples of these phenomena are shown in Figure 12.

**Figure 13:**
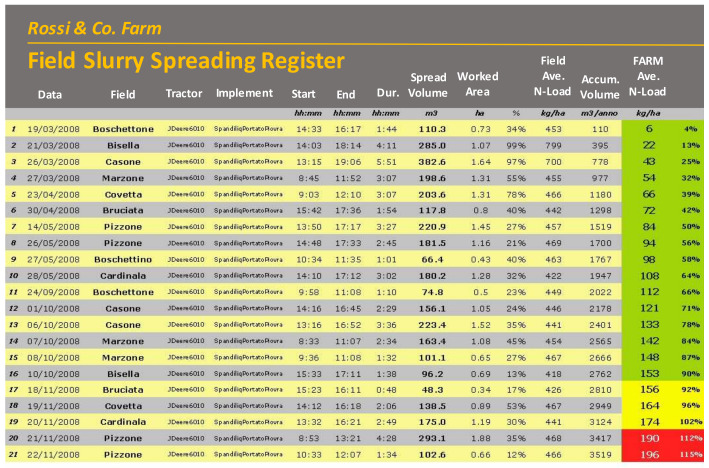
Example of yearly report obtained by combining information from different sources of automated operational monitoring applications (slurry levels in tank, and field slurry spreading registers, as part of a FORK monitoring system). The farm at hand, being in a vulnerable area, is subject to a N-load limitation of 170 kg/(h·year). Such integrated system provides early warning when limit values are going to be reached.

For spreading operations, operational monitoring solutions similar to the already mentioned FORK systems can be applied, equipped both with identification system for automatic recognition of the slurry tank used, and with GNSS receivers (of medium-low precision/accuracy) necessary to recognize the slurry collection points and the fields on which they are then distributed. Information from FORK and tank monitoring systems, once integrated, can provide summary reports similar to the one shown in Figure 13. Here, the nitrogen load balances actually distributed are also indicated, thus even requiring measurements/estimations of the slurry N-contents.

To this aim, very often it is considered sufficient to refer to tabular average data, possibly updated through periodic chemical analyses on a sample basis. Different and more sophisticated devices could be able to provide estimates of N concentrations also through indirect measurements (conductivity, absorption in the NIR band [58,59,60,61]), but their application is more expensive and not easy to manage (in this case, we enter the domain of MPI-2, since they involve measurements on the properties of animal by-products). The technological choices related to the definition of the overall reliability of the monitoring system always remain dependent on the decisional objectives of the farm managers.

A variety of other agricultural practices show the same features of the last integrated monitoring system here discussed [62,63]. Generally, their actual and final usefulness is expressed in control actions that typically require the integration between several independent systems and a variety of interpretative procedures (inference engines). As we saw, their reference domain is always confined in the MPI-3, with performances that, however, should be certified by appropriate procedures in order to make transparent the levels of reliability of the monitoring carried out in compliance with both the current regulations and the decisional objectives of the farmers.

## 5. Discussion and Conclusions

The application areas of the SA are constituted by complex systems in which the measurement conditions of a given physical quantity are often far from the “controlled environment” requirements governing the traditional domain of metrology. In this domain, in fact, it is usually possible to operate in an “ideal” context in which the measurement instruments and procedures are based on aspects able to guarantee very high accuracy, with performances attributable only to the reliability of the instruments themselves. Even any noise phenomenon inducible from the external environment (physical or human) can be largely modulated here, as well as exploitable to determine the influence of the noise itself on the quality of the final measurement. These are fundamental requirements for every certification activity on the performance of any direct or indirect measuring instrument, normally based on quality levels of scientific and technological knowledge typical of the MPI-1 and MPI-2 macro-domains (although with more fuzzy aspects for the latter, for the non-linear behavior of biological processes). Both focus on the importance of measurement “in itself”, regardless of the use that could be made of it within a decision-making process.

For all of the above, however, in the case of SA we have that:

- it operates in an entrepreneurial environment closely related to the MPI-3 macro-domain, where the measures must support information to be used for solving decision-making problems that require different accuracies depending on the hierarchical level that must manage the decision itself; precise measures are not sought, but rather functional and satisfactory information with respect to a given objective; not infrequently, this objective must also meet requirements imposed by regulatory frameworks, to prove compliance of products and processes with administrative rules or best practice protocols;
- the information rarely originates from data obtained only from a single measuring instrument; these are usually indirect measurements resulting from the integration of several measuring systems (in turn derived from the combination of individual instruments);
- measurements are often not limited to the use of sensors (simple or complex sensors); by definition, a sensor is a *power transducer* that transforms the value of a physical or chemical quantity associated with real world phenomena into an intelligible message; in management environments, where SA is involved, it is often essential to recognize even the agents participating in a given event; hence the need to be able to integrate in data acquisition devices also *identification systems*, which operate on completely different logical and physical principles (comparison systems with reference elements) with respect to sensors; in their case, the concept of measurement accuracy is replaced by the concept of *recognition accuracy* and the verification of their performance requires its own approaches and metrics;
- many monitoring activities must be carried out under dynamic conditions, since the measurement/identification systems are mounted on moving means; this necessity, which in itself already complicates the measurement conditions, normally requires that every observation on the real world is also integrated with a position data for its instantaneous localization; hence, the need for *positioning systems*, which are neither sensors nor identification systems; in the case of GNSS, they are rather technologically similar to radio wave transmission systems and therefore require their own approaches for the verification of related performances;
- thus, the “final measurements” often derive from the integration of the responses from very different technological components; for their interpretation, it is almost always mandatory to use customized inference engines, often implemented on the specific needs of each measurement system, or the need to integrate measurements from multiple systems. However, the modeling interpretation is an integral part of the monitoring processes of SA and their behavior must be verified (validated) in the same way as any other technological hardware component.

These are also technological requirements of Ind-4.0, which pushes on hyper-connectivity and cyber-physical systems, with application areas that pose the same challenges as above (e.g., predictive maintenance). Here also, similarly to SA, there is the need to frame the concept of “information precision” through new approaches, providing new operational indications for the certification of the reliability of information on the whole decisional chain. Moreover, it is precisely on the certification front that the next technological challenges should be played out; challenges that will possibly have to be met jointly by manufacturers and researchers. Today, many technologies able to address all or part of the difficulties mentioned above are already available. In many cases, however, these are niche technologies or even prototypes, with solutions in continuous evolution, with respect to which possible users remain disoriented with difficulty in understanding the real benefits that the company/farm could achieve from their adoption. This is especially true for applications that lead to the management domains of MPI-3, especially if there is a need to rely on integrated information systems (IIS) for the inclusion of multiple processes or production sectors.

The core of the challenge, therefore, becomes how to certify a technological system articulated on several subsystems, which in turn consist of several devices with different operating principles, including also software components with functions at various levels (inference engines in data acquisition systems or decision support tools). In this sense, new certification approaches in a MPI-3 key must be able to highlight:

1. the intervention objectives and related decision-making strategies, seeking-where possible-to provide measurability criteria for the objectives;
2. the minimum degree of efficacy allowed, always in relation to the decision-making objectives;
3. the list of information necessary for the decision-making process, with the relative degree of reliability required (overall tolerance);
4. for each item of information: specify the requirements for the measurement equipment (in terms of accuracy and precision) and for the related inference engines;
5. the test modes in controllable environments for each measuring equipment, taking into account the different technological behavior of each component (sensor, identification system, positioning system);
6. the validation methods for the most relevant interpretation procedures.

Many challenges can be opened already from the realization of the previous points 5 and 6, thinking about the possibility of constructing physical scale models to allow the repeatability of tests in controlled environments, certainly suitable for the integration of multiple measurement systems to be tested/certified even under prolonged operating conditions over time. All of this, following similar approaches, for example, to the above-mentioned RotoGPS, and even looking for shared approaches among research groups in order to reach standard reference solutions, useful for the development of the various application areas of SA.

In conclusion, the new approaches in the certification of SA application systems will have to guarantee instrumental and methodological consistency throughout the decision-making chain, ensuring a strong consistency between measures and decision-making objectives.

## Figures and Tables

**Table 1:** Proposal for a possible classification of the evolutionary phases of farm technological innovations (periods derived mainly based upon the Italian situation).

Agriculture	Period	Name	Description
**0.0**	Until 1920	**Tradition and Ruralism**	Prevalent use of manual labor force and animal traction; progressive transit towards motorized traction in the final phase.
**1.0**	1920–1960	**Motorization**	Introduction and diffusion of innovations in the tractor sector (diesel engines, hydraulic circuits, three-point linkage, “invention” of tires); the use of field manual labor continues to be prevalent; in Italy, this phase has been prolonged by autarkic rural policies wanted by Fascism; acceleration of the transition after World War II with the electrification of agricultural areas.
**2.0**	1960–1980	**Mechanization**	Tractor refinement with progressive increase of its nominal power and performances; strong development of the operating machines introduced in all farm production areas; fast replacement of the manual labor force and exodus from the countryside; increase of the primary yields also, thanks to the innovations in the genetic field and in the chemical industry.
**3.0**	1980–2000	**Humanism and Electronics**	Improvement of mechanized systems with greater attention to the man-machine relationship (ergonomics and safety); introduction of electronic control systems on board tractors and first fixed-point process automation solutions; first attempts to digitalize farm management, never fully and widely consolidated, except in specific sectors such as animal husbandry (especially in dairy farms).
**4.0**	After 2000	**PF, Traceability and Cyber Physical Systems** **(Smart Agriculture)**	Consolidation of electronics and automation in all farm sectors, with a strong focus on the automation of mobile-point processes (site-specific control); diffusion of sensors in monitoring activities and on board tractor positioning systems; communication protocols between devices (CAN, ISOBUS, Wireless networks, Wi-Fi, Bluetooth, etc.); experiences in computerization and use of integrated information systems, especially in large farms; process connectivity and M2M communications; IoT and IoS; cloud and fog computing; proposals for proprietary solutions of farm information systems by major brands of the farm machinery market; product and process certification aimed at traceability systems

Abbreviations: Internet of Things (IoT) and Internet of Services (IoS); Precision Farming (PF); Controller Area Network (CAN); ISOBUS: serial network for control and communication on tractors, defined by ISO 11783; Machine-to-Machine Communications (M2M).

## Abbreviations

Agr-4.0	Agriculture 4.0
CEP95	Radius of the Circle Error Probable, where 95% of the GNSS fixings would fall in
CNC	Computer Numerical Control
CPS	Cyber physical systems
DGNSS	Differential Global Navigation Satellite System
ERP	Enterprise Resource Planning
FORK	Field Operation Register Keeping
GNSS	Global Navigation Satellite System
HDOP	Horizontal Dilution of Precision
ICT	Information & Communication Technology
IIS	Integrated Information System
Ind-3.0	Industry 3.0
Ind-4.0	Industry 4.0
IoE	Internet of Everything
IoT	Internet of Things
IT	Information Technology
KM4.0	Knowledge Management 4.0
ML	Machine Learning
M2M	Machine to Machine Communications
MPI	Macrodomain of Prevailing Interest
MSS	Management Support Systems
NIR	Near Infra-Red band
NOP	Not Observable part of a system
OP	Observable part of a system
PF	Precision Farming
SA	Smart Agriculture
SDSS	Strategic Decision Support Systems
UML	Unified Modeling Language
US NRC	National Research Council of United States
WSN	Wireless Sensor Networks

## References

[1] Aubert B.A., Schroeder A., Grimaudo J. (2012). IT as enabler of sustainable farming: An empirical analysis of farmers’ adoption decision of precision agriculture technology. Decis. Support Syst..

[2] Cox S. (2002). Information technology: The global key to precision agriculture and sustainability. Comput. Electron. Agric..

[3] Wan J., Cai H., Zhou K. Industrie 4.0: Enabling technologies. Proceedings of the 2015 International Conference on Intelligent Computing and Internet of Things.

[4] US NRC (1997). Precision Agriculture in the 21st Century: Geospatial and Information Technologies in Crop Management. Comm. on Assessing Crop Yield, Site- Specific Farming, Information Systems and Res. Opportunities, Board on Agric.

[5] Rao M.S., Suresh Babu E., Siva Naga Raju P., Kavati I. (2019). Smart agriculture: Automated controlled monitoring system using internet of things. Int. J. Recent Technol. Eng..

[6] Gurnule P.V. (2019). Economical smart agriculture monitoring system. Int. J. Recent Technol. Eng..

[7] Jin X.-B., Yang N.-X., Wang X.-Y., Bai Y.-T., Su T.-L., Kong J.-L. (2020). Hybrid deep learning predictor for smart agriculture sensing based on empirical mode decomposition and gated recurrent unit group model. Sensors.

[8] Ciruela-Lorenzo A.M., Del-Aguila-Obra A.R., Padilla-Meléndez A., Plaza-Angulo J.J. (2020). Digitalization of agri-cooperatives in the smart agriculture context. Proposal of a digital diagnosis tool. Sustainability.

[9] Shamim S., Cang S., Yu H., Li Y. Management approaches for Industry 4.0: A human resource management perspective. Proceedings of the IEEE Congress on Evolutionary Computation (CEC).

[10] Rodríguez-Molano J.I., Contreras-Bravo L.E., Rivas-Trujillo E., Rocha Á., Guarda T. (2018). Industry Knowledge Management Model 4.0. Proc. of the International Conference on Information Technology & Systems. Advances in Intelligent Systems and Computing.

[11] Chiang M., Zhang T. (2016). Fog and IoT: An Overview of Research Opportunities. IEEE Internet Things J..

[12] Baccarelli E., Vinuenza Naranjo P.G., Shojafar M., Abawajy J.H. (2017). Fog of everything: Energy-efficient networked computing architectures, research challenges, and a case study. IEEE Access.

[13] Morais P., Silva N., Mendes J., Adão T., Pádua L., López-Riquelme J.A., Pavón-Pulido N., João Sousa J., Peres E. (2019). Mysense: A comprehensive data management environment to improve precision agriculture practices. Comput. Electron. Agric..

[14] Sourav K., Arijit S., Ruhul A. (2019). An overview of cloud-fog computing: Architectures, applications with security challenges. Secur. Priv..

[15] Tsipis A., Papamichail A., Koufoudakis G., Tsoumanis G., Polykalas S.E., Oikonomou K. (2020). Latency-Adjustable Cloud/Fog Computing Architecture for Time-Sensitive Environmental Monitoring in Olive Groves. AgriEngineering.

[16] Candanedo I.S., Nieves E.H., González S.R., Martín M.T.S., Briones A.G. (2018). Machine learning predictive model for industry 4.0. Commun. Comput. Inf. Sci..

[17] Bonnell J. (2019). The importance of predictive, maintenance. Weld. J..

[18] Chuang S.-Y., Sahoo N., Lin H.-W., Chang Y.-H. (2019). Predictive maintenance with sensor data analytics on a Raspberry Pi-based experimental platform. Sensors.

[19] Short M., Twiddle J. (2019). An industrial digitalization platform for condition monitoring and predictive maintenance of pumping equipment. Sensors.

[20] Xie N.F., Wang W.S., Yang Y. Ontology-based Agricultural Knowledge Acquisition and Application. Proceedings of the 2nd IFIP Int. Conference Computer and Computing Technologies in Agriculture.

[21] Kim J.Y., Lee C.G., Baek S.H., Rhee J.Y. (2015). Open farm information system data-exchange platform for interaction with agricultural information systems. Agric. Eng. Int. CIGR J..

[22] Mazzetto F., Gallo R., Riedl M., Sacco P. (2019). Proposal of an ontological approach to design and analyse farm information systems to support Precision Agriculture techniques. IOP Conf. Ser. Earth Environ. Sci..

[23] Methlie L. (1980). Data management for decision support systems. Database.

[24] Westrup C., Orlikowski W.J., Walsham G., Jones M.R., DeGross J. (1996). Transforming organizations through systems analysis: Deploying new techniques for organizational analysis in Information Systems development. Information Technology and Changes in Organizational Work.

[25] Drucker P.F. (2006). Knowledge-worker productivity, the biggest challenge. IEEE Eng. Manag. Rev..

[26] Rae R.H., Tan K.H. Working knowledge: How to manage and retain contract workers knowledge. Proceedings of the 22nd International Conference on Production Research ICPR.

[27] Anthony R.N. (1965). Planning and Control: A Framework for Analysis.

[28] Sousa M.J., Dias I., Cruz R., Caracol C. (2016). Information Management Systems in the Supply Chain.

[29] Vansteenkiste G.C., Sydow A., Tzafestas S.G., Vichnevetsky R. (1988). New Simulation Approaches to Ill-Defined Systems. Systems Analysis and Simulation I. Advances in Simulation.

[30] Kops S., Vangheluwe H., Claeys F., Vanrolleghem P., Yuan Z., Vansteenkiste G. (1999). The Process of Model Building and Simulation of Ill-Defined Systems: Application to Wastewater Treatment. Math. Comput. Model. Dyn. Syst..

[31] Ping J.L., Dobermann A. (2005). Processing of yield map data. Precis. Agric..

[32] Pierce F.J., Nowak P. (1999). Aspects of Precision Agriculture. Adv. Agron..

[33] Stafford J. (1999). GPS in Agriculture—A Growing Market. J. Navig..

[34] Pérez Ruiz M., Upadhyaya S. (2012). GNSS in Precision Agricultural Operations. New Approach of Indoor and Outdoor Localization Systems.

[35] Landonio S. (2018). Personal communication.

[36] Mazzetto F., Azzoli G., Calcante A., Castelli G., Blandini G., Manetto R. (2005). ROTOGPS: Uno strumento per la misura di precisione e accuratezza di ricevitori GPS (ROTOGPS: A tool for measuring accuracy and precision of GPS receivers). L’ingegneria Agraria Per lo Sviluppo Sostenibile Dell’area Mediterranea.

[37] Azzoli G. (2004). II ROTOGPS: Uno Strumento Per La Valutazione Delle Prestazioni Di Ricevitori Gps in Ambienti Agricoli (The ROTOGPS: A Tool for Evaluating the Performance of GPS Receivers in Agricultural Environments). Master’s Thesis.

[38] Mazzetto F., Gallo R., Importuni P., Petrera S., Sacco P. (2017). Automatic filling of field activities register, from challenge into reality. Chem. Eng. Trans..

[39] Mazzetto F., Calcante A. (2011). Highly automated vine cutting transplanter based on DGNSS-RTK technology integrated with hydraulic devices. Comput. Electron. Agric..

[40] Keicher R., Seufert H. (2000). Automatic guidance for agricultural vehicles in Europe. Comput. Electron. Agric..

[41] Vega A., Córdoba M., Castro-Franco M., Balzarini M. (2019). Protocol for automating error removal from yield maps. Precis. Agric..

[42] Abramov N.V. (2019). Yield mapping using satellite navigation systems. IOP Conf. Ser. Mater. Sci. Eng..

[43] Luck J.D., Fulton J.P. (2014). Best Management Practices for Collecting Accurate Yield Data and Avoiding Errors during Harvest. Univ. Neb. Ext. Linc. NE.

[44] Birrell S.J., Sudduth K.A., Borgelt S.C. (1996). Comparison of sensors and techniques for crop yield mapping. EC2004. Comput. Electron. Agric..

[45] Stafford J.V., Ambler B., Lark R.M., Catt J. (1996). Mapping and interpreting the yield variation in cereal crops. Comput. Electron. Agric..

[46] Louhaichi M., Young W.C., Johnson E.D. (2013). Reliability of Yield Mapping System for Estimating Perennial Ryegrass Seed Yield. Aust. J. Basic Appl. Sci..

[47] Panten K., Haneklaus S., Schnug E. (2002). Spatial accuracy of online yield mapping. Landbauforsch. Volkenrode.

[48] Arslan S., Colvin T.S. (2002). Grain yield mapping: Yield sensing, yield reconstruction, and errors. Precis. Agric..

[49] Griffin T.W., Dobbins C.L., Vyn T.J., Florax R.J.G.M., Lowenberg-Deboer J.M. (2008). Spatial analysis of yield monitor data: Case studies of on-farm trials and farm management decision making. Precis. Agric..

[50] Marchant B., Rudolph S., Roques S., Kindred D., Gillingham V., Welham S., Coleman C., Sylvester-Bradley R. (2019). Establishing the precision and robustness of farmers’ crop experiments. Field Crop. Res..

[51] Chen Y., Wang X., Zhao C. Prescription Map Generation Intelligent System of Precision Agriculture Based on Web Services and WebGIS. Proceedings of the 2009 International Conference on Management and Service Science.

[52] Rahelizatovo N.C., Gillespie J.M. (2004). Factors influencing the implementation of best management practices in the dairy industry. J. Soil Water Conserv..

[53] Sacco P., Gallo R., Mazzetto F. (2019). Data analysis and inference model for automating operational monitoring activities in Precision Farming and Precision Forestry applications. IOP Conf. Ser. Earth Environ. Sci..

[54] Calcante A., Mazzetto F. (2014). Design, development and evaluation of a wireless system for the automatic identification of implements. Comput. Electron. Agric..

[55] Gallo R., Carabin G., Vidoni R., Sacco P., Mazzetto F. (2018). Solutions for the automation of operational monitoring activities for agricultural and forestry tasks. Bodenkultur.

[56] Ristorto G., Mazzetto F., Guglieri G., Quagliotti F. (2015). Monitoring performances and cost estimation of multirotor Unmanned Aerial Systems in precision farming. Int. Conf. Unmanned Aircr. Syst..

[57] Mazzetto F., Sacco P., Calcante A. (2012). Algorithms for the interpretation of continuous measurement of the slurry level in storage tanks. J. Agric. Eng..

[58] Saeys W., Mouazen A.M., Ramon H. (2005). Potential for onsite and online analysis of pig manure using visible and near infrared reflectance spectroscopy. Biosyst. Eng..

[59] Provolo G., Martínez-Suller L. (2007). In situ determination of slurry nutrient content by electrical conductivity. Bioresour. Technol..

[60] Tamburini E., Castaldelli G., Ferrari G., Marchetti M.G., Pedrini P., Aschonitis V.G. (2015). Onsite and online FT-NIR spectroscopy for the estimation of total nitrogen and moisture content in poultry manure. Environ. Technol..

[61] Perricone V., Costa A., Calcante A., Agazzi A., Savoini G., Sesan E., Chiara M., Tangorra F.M. TMR mixer wagon real time moisture measurement of animal forages. Proceedings of the 2019 IEEE International Workshop on Metrology for Agriculture and Forestry (MetroAgriFor).

[62] Kaloxylos A., Groumas A., Sarris V., Katsikas L., Magdalinos P., Antoniou E., Politopoulou Z., Wolfert S., Brewster C., Eigenmann R. (2014). A cloud-based farm management system: Architecture and implementation. Comput. Electron. Agric..

[63] Bauerdick J., Piringer G., Gronauer A., Kral I., Bernhardt H. (2017). Precision Grassland Farming—An overview of research and technology (Precision Grassland Farming—Ein überblick über Forschung und Technik). Lecture Notes in Informatics (LNI), Proceedings Series of the Gesellschaft fur Informatik (GI).

